# Phosphate Binder Impairs Levothyroxine Absorption in a Hypothyroid Patient With End-Stage Renal Disease

**DOI:** 10.7759/cureus.71551

**Published:** 2024-10-15

**Authors:** Michelle Ko, Rakhee Barai, Gregory Brent

**Affiliations:** 1 Medicine, David Geffen School of Medicine, University of California Los Angeles, Los Angeles, USA

**Keywords:** autoimmune hypothyroidism, chronic hemodialysis, end-stage renal disease (esrd), levothyroxine malabsorption, levothyroxine therapy, liquid levothyroxine

## Abstract

End-stage renal disease (ESRD) patients have an increased incidence of hypothyroidism, and those with serum thyroid stimulating hormone (TSH) levels above the reference range have excess mortality, increased cardiovascular disease, impaired health-related quality of life, and altered body composition. We report a patient with ESRD on chronic hemodialysis and Hashimoto’s disease, who is on chronic levothyroxine therapy. Despite a high levothyroxine dose of 2.12 mcg/kg and regular adherence, the patient had elevated TSH levels and a pattern of erratic TSH levels. The patient was on the phosphate binder, sevelamer, which has been associated with reduced levothyroxine absorption. The patient was switched to a liquid levothyroxine preparation at the same dose, and after two months, free thyroxine levels normalized and TSH levels improved. The implications of hypothyroidism in patients with ESRD are discussed, along with approaches to managing erratic serum TSH levels and the use of liquid levothyroxine preparations to improve serum TSH levels in patients taking medications that reduce absorption.

## Introduction

Hypothyroidism is a common endocrine disorder that affects as much as 5% of the population [1,2]. A much higher fraction of patients on hemodialysis, approximately 10-25%, have abnormalities in thyroid function tests, with the majority being hypothyroid. Many of these patients are on levothyroxine therapy [3-5]. Several drugs are known to interfere with the absorption of levothyroxine, which is important to be aware of in dialysis patients, who are typically on a multidrug regimen [1,4]. Appropriate adjustment of the levothyroxine dose to achieve TSH concentrations within the reference range is critical for patients with chronic kidney disease (CKD). Multiple studies have shown that an elevated serum TSH level in patients on hemodialysis is associated with excess mortality, increased cardiovascular disease, impaired health-related quality of life, and altered body composition, compared to those with reference range TSH levels [4]. Here, we report the case of a 63-year-old man with end-stage renal disease (ESRD) on hemodialysis and chronic hypothyroidism due to Hashimoto’s disease. He had a persistently elevated TSH, despite taking a high dose of levothyroxine based on his weight (2.12 mcg/kg), likely due to impaired levothyroxine absorption associated with the phosphate binder, sevelamer [6].

## Case presentation

A 63-year-old man with a history of Hashimoto’s disease and ESRD on hemodialysis three times per week presented for hypertensive urgency prior to a hemodialysis session. He also had one month of fatigue, constipation, and dry skin, and on laboratory evaluation was found to have low serum free thyroxine (FT4) and high serum TSH levels. The patient had a greater than 40-year history of hypothyroidism as well as several chronic medical conditions (Table 1).

**Table 1:** Chronic medical conditions

Medical Conditions
End-Stage Renal Disease (ESRD) on Hemodialysis
Hashimoto’s Disease, Chronic
Bipolar Disorder Schizoaffective Disorder
Chronic Obstructive Pulmonary Disease
Heart Failure With Mildly Reduced Ejection Fraction
Hyperlipidemia
Hypertension
Neurogenic Bladder
Post-Traumatic Stress Disorder
Remote History Polysubstance Use Disorder

**Figure 1:**
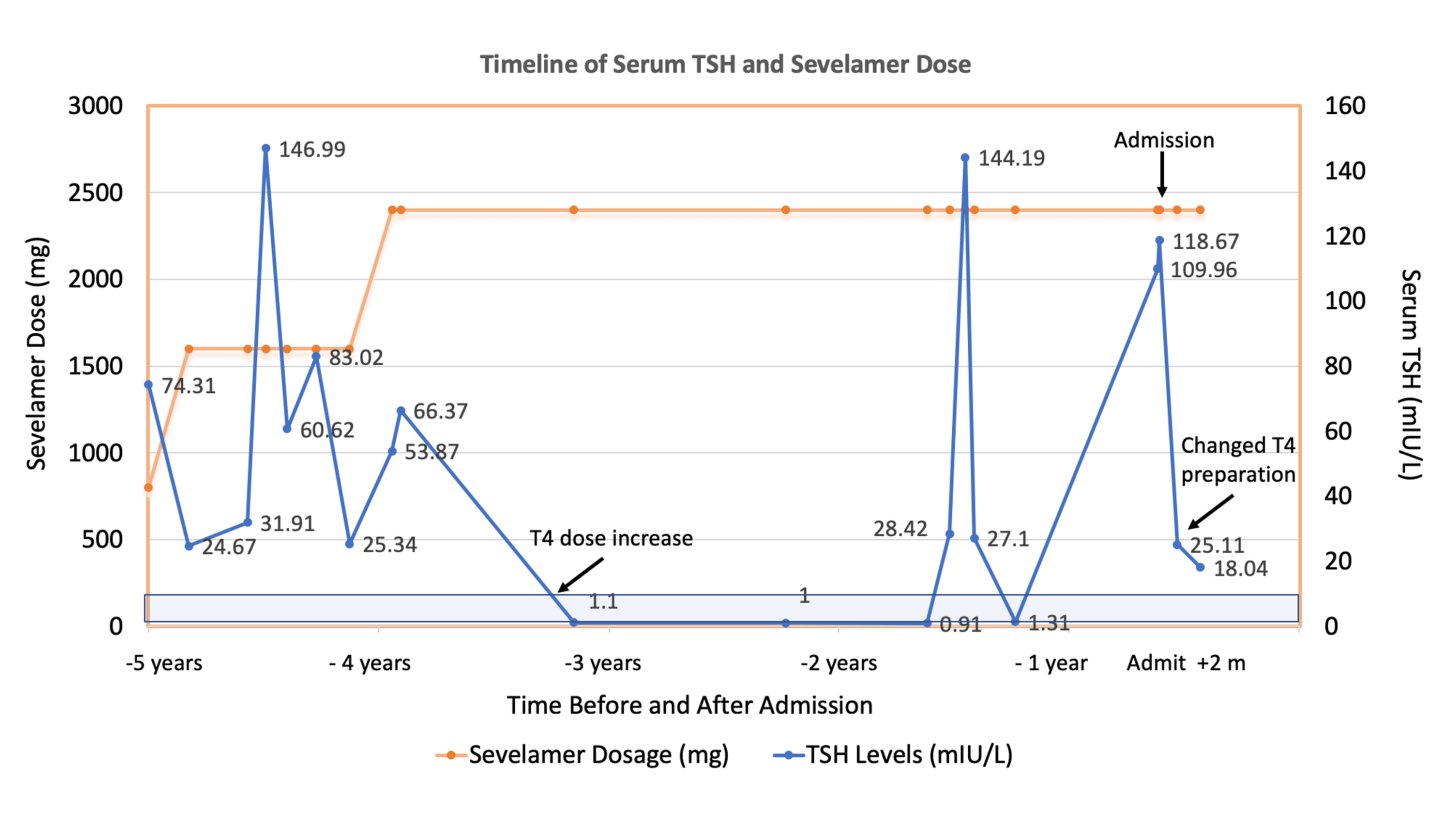
Graph showing patient’s serum TSH levels and sevelamer dose The serum TSH level (mIU/L) and sevelamer dose (mg) were taken three times per day, shown at time points during the period of five years prior to admission (shown by arrow) and two months (+2m) after discharge. The levothyroxine (T4) dose was increased from 150 mcg per day to 175 mcg per day at the time point shown by the arrow (T4 dose increase). The change in T4 preparation on discharge from levothyroxine tablets to gel capsules is shown by an arrow (changed T4 preparation). The sevelamer dose is shown with an orange line and the serum TSH level with a blue line. The shaded blue area shows the TSH reference range. TSH, thyroid stimulating hormone

He denied a history of goiter, thyroid nodules, or radiation to the neck. He denied recent illnesses or any recent changes in medication, other than an increase in carvedilol dose to 25 mg twice per day. He had been on levothyroxine at varying doses, based on a review of his medical records, with periods of erratic serum FT4 and TSH measurements (Figure 1). Relevant medications included sevelamer carbonate (2400 mg three times per day) and levothyroxine (175 mcg daily in the morning), as well as medications for his various chronic medical conditions (Table 2).

**Table 2:** Outpatient medications

Medication	Dose
Levothyroxine	175 mcg daily
Sevelamer	2400 mg three times per day
Albuterol	Four times daily as needed
Atorvastatin	40 mg daily
Calcitriol	1 mcg three times per week
Carvedilol	25 mg two times per day
Lisinopril	20 mg daily
Multivitamins (Renal)	1 tablet daily
Nifedipine	90 mg daily
Olodaterol/tiotropium	2 puffs daily, as needed
Ondansetron	2 mg daily, as needed
Oxcarbazepine	600 mg two times per day
Oxycodone	5 mg every 4 hours, as needed

Although the erratic thyroid studies were previously attributed to medication nonadherence, the patient reported adherence to morning dosing of levothyroxine for the past two years and was taking levothyroxine on an empty stomach. The patient reported taking levothyroxine daily at 4 AM with water and did not take any other medications or eat breakfast until 8 AM. 

In the emergency department, the patient was hypertensive with a blood pressure of 163/106 and bradycardic with a heart rate of 60 beats per minute. Other vital signs were within normal limits with a temperature of 36.3C, respiratory rate of 16 breaths/minute, and oxygen saturation of 97% on room air. His height was 182.9 cm, weight was 82.33 kg, and body mass index (BMI) was 24.7 kg/m^2^. The patient was alert and oriented to person, place, and time and was not in any acute distress. Physical examination was significant for the findings of dry skin and hair and slowed relaxation phases of his deep tendon reflexes. He did not have neck tenderness, thyroid enlargement, or a palpable thyroid nodule. Laboratory studies on admission and one and two months after discharge are shown in Table 3 and Figure 1.

**Table 3:** Laboratory investigations

Laboratory Study	Admission	1 Month Post-discharge	2 Months Post-discharge	Reference Range
Thyroid Stimulating Hormone (TSH)	109.96 mIU/L	25.11 mIU/L	18 mIU/L	0.55-4.78
Free Thyroxine (FT4)	0.53 ng/dL	0.59 ng/dL	0.76 ng/dL	0.66-1.73
Thyroid Peroxidase Antibody (TPO Ab)	97.9 IU/mL	-	-	-

The patient was admitted for treatment and monitoring of elevated blood pressure. Thyroid studies showed overt hypothyroidism with elevated TSH, reduced Free T4, and elevated TPO antibodies, consistent with underlying Hashimoto's thyroiditis. The patient was given intravenous levothyroxine 140 mcg (calculated at 80% of his outpatient levothyroxine dose of 175 mcg) due to concern for reduced absorption from the enteral route from gut edema, which is associated with more severe hypothyroidism [1]. The patient stated that he had been taking his medication daily on an empty stomach, in the morning. The patient had been on sevelamer for several years and had periods of time of adequate levothyroxine replacement with reference range serum FT4 and TSH, but predominantly had erratic TSH levels. The potential to normalize TSH with a liquid levothyroxine preparation was considered. On discharge, the patient was switched from the levothyroxine tablets he was previously on to the liquid capsule formulation, Tirosint, at a dose of 175 mcg per day. Thyroid function tests were collected one month after discharge on this regimen, which demonstrated improvement, with a reduction in TSH and an increase in FT4. Two months after discharge, his TSH was further reduced toward the reference range and FT4 normalized (Table 3 and Figure 1). 

## Discussion

Drugs have multiple effects on the thyroid and on thyroxine therapy [7]. Based on the usual weight-based levothyroxine dosing of 1.6-1.8 mcg/kg per kg body weight, one would expect the patient (82.33 kg) to require a daily levothyroxine dose in the range of 131-148 mcg to maintain euthyroidism, if he was consistently taking his medication and absorbing the dose appropriately [1,2]. While on a levothyroxine dose over this range, 175 mcg (2.12 mcg/kg), the patient experienced erratic, often markedly elevated levels of TSH, suggesting reduced absorption of levothyroxine. The patient was receiving oxcarbazepine (Table 2), which has been associated with reductions in serum FT4, but does not affect TSH levels, although it may have been a contributing factor [8]. The primary considerations for patients with erratic TSH levels are inconsistent medication adherence or reduced absorption from interfering medications, as well as conditions associated with malabsorption, such as celiac disease [1]. There have been reported differences in absorption based on the levothyroxine manufacturer and the potential for degradation of tablets if stored improperly. Still, these are less likely to cause this magnitude of TSH elevation.

Disruption of levothyroxine absorption may occur at various levels and an increasing list of medications and conditions have been recognized with this effect [1,9]. Medications that increase the gastric pH, including proton pump inhibitors and histamine H2-receptor antagonists, consistently reduce absorption. Medications may reduce mucosal transport by interfering with the organic anion transporting polypeptide, including ciprofloxacin and rifampin. Finally, certain medications may directly interfere with levothyroxine, including calcium and iron supplements, as well as phosphate binders [9]. Phosphate binders, especially sevelamer, are associated with higher TSH levels in dialysis patients [10]. Phosphate binders such as inorganic salts (aluminum hydroxide, calcium salts, and magnesium hydroxide) typically release ions to trap dietary phosphate in the gut. Sevelamer is a cationic hydrogel with multiple amine groups that become protonated in the GI tract and bind nonspecifically with anionic phosphate and other anion substances such as levothyroxine, interfering with their absorption [6]. An observational study of hemodialysis patients on levothyroxine and phosphate binders (n=67) found that patients on calcium carbonate and sevelamer hydrochloride had significantly higher TSH levels compared to those being treated only with calcium acetate [10]. There have been several case reports that support these findings, with cases of patients experiencing erratic TSH levels while taking sevelamer [6].

A variety of approaches have been proposed to evaluate patients with erratic levels of TSH or persistently elevated serum TSH, despite adequate or even elevated doses of levothyroxine [1,2,7]. If these findings persist after confirming that the patient is taking levothyroxine on an empty stomach and without interfering medications, possible approaches include supervised weekly dosing or a formal levothyroxine absorption test [11]. An approach to treating patients with levothyroxine malabsorption is to switch to thyroid supplementation formulations that are better absorbed, such as liquid solutions or soft gelatin capsules [8,12]. These formulations do not contain gluten and are, therefore, also effective in patients with malabsorption due to celiac disease. A recent absorption study showed that liquid thyroxine preparations are well absorbed in patients taking proton pump inhibitors [13].

A contributing factor to our patient’s elevated and erratic TSH levels while on high-dose levothyroxine was considered to be due to impaired levothyroxine absorption from concurrent sevelamer treatment. Although medication nonadherence was considered, the patient reported taking levothyroxine with the appropriate regimen for the past two years. The patient had a reference range FT4 two months after the switch to a liquid preparation. The TSH remained above the reference range at two months; however, according to a recent study, normalization of TSH after high elevation in overt hypothyroidism takes a median of 8.9 months, with a range of 7.6 to 10.2 months [14]. Although interference from phosphate binders is reported in the nephrology literature, it has not been noted frequently in the general medical literature [7].

## Conclusions

Clinicians should recognize the conditions and medications that can alter levothyroxine absorption in hypothyroid patients, especially those on hemodialysis who are taking multiple medications, as significant consequences can arise when TSH levels are not within target ranges. Multiple factors may contribute to the inability to normalize serum TSH in hypothyroid patients, including levothyroxine dose, preparation, and adherence. A large observational study of patients with hypothyroidism on hemodialysis, however, found that sevelamer was associated with high TSH levels, more so than calcium acetate. Although not studied specifically in this population, liquid and soft gelatin capsule formulations of levothyroxine have been shown to improve absorption and should be considered as an approach to normalize levothyroxine absorption in select patients.
